# Green Foot Discoloration Secondary to Pseudomonas aeruginosa in an Elderly Male

**DOI:** 10.7759/cureus.112970

**Published:** 2026-07-19

**Authors:** Jasmin K Bhullar

**Affiliations:** 1 Department of Internal Medicine, University of North Carolina Health Southeastern, Lumberton, USA

**Keywords:** cellulitis, chronic venous insufficiency, elderly, green discoloration, nursing facility, pseudomonas aeruginosa, pyocyanin, wound colonization

## Abstract

Green discoloration of the skin is an uncommon clinical finding that can cause significant alarm among patients and caregivers. We present the case of a 92-year-old man residing in a nursing facility with a past medical history significant for hypertension, type 2 diabetes mellitus, stage 3a chronic kidney disease, chronic venous insufficiency, prior left ankle surgery with retained internal fixation hardware, and a history of deep venous thromboses who presented to the ED with acute-onset green discoloration of his left foot. The discoloration was first noticed by the nursing staff at his facility. The patient denied pain, fever, or chills. Physical examination revealed green discoloration of the plantar surface of the left foot, bilateral lower extremity edema with stasis dermatitis, chronic skin hyperkeratosis, onychomycosis, and a superficial skin laceration on the dorsal aspect of the foot. Laboratory evaluation demonstrated elevated blood urea nitrogen and creatinine levels consistent with acute kidney injury and a normal lactate level. Radiography showed diffuse soft tissue edema without fracture. The Infectious Diseases service was consulted. Clinical assessment determined that the green discoloration was attributable to *Pseudomonas aeruginosa* colonization, with secondary superimposed cellulitis arising from excoriations caused by ill-fitting footwear. The patient was treated with IV cefepime followed by step-down therapy with oral cephalexin and doxycycline, along with acetic acid soaks and wound care. Blood cultures remained negative. The patient demonstrated clinical improvement and was discharged to his nursing facility with wound care follow-up. This case highlights the clinical challenge of distinguishing *Pseudomonas* colonization from invasive infection in elderly patients with chronic skin changes and underscores the importance of a multidisciplinary approach involving infectious diseases and wound care.

## Introduction

*Pseudomonas aeruginosa* is a Gram-negative bacillus recognized as a significant opportunistic pathogen, particularly in vulnerable hosts [1,2]. The organism produces several virulence factors, most notably pyocyanin, a redox-active phenazine pigment responsible for the characteristic blue-green coloration associated with *Pseudomonas*-related skin conditions [1]. This pigment can lead to the clinical finding of green skin discoloration. Pyocyanin biosynthesis is regulated by quorum-sensing networks involving the *phz1 *and *phz2 *operons, and its visible accumulation on skin surfaces may reflect a significant bacterial burden [3]. However, skin discoloration remains a clinical observation rather than a definitive diagnostic marker, as microbiological confirmation of *Pseudomonas* is not always performed or required. Beyond its role as a pigment, pyocyanin induces oxidative stress in host cells, inhibits cellular proliferation, and impairs tissue repair [4].

Cutaneous manifestations of *P. aeruginosa* span a broad clinical spectrum. In immunocompetent hosts, common presentations include green nail syndrome (chloronychia), toe web infections, hot tub folliculitis, and hot-foot syndrome [2]. These conditions are generally mild, self-limited, and amenable to local or topical therapy. In contrast, immunocompromised or debilitated patients may develop more severe manifestations, including ecthyma gangrenosum, necrotizing soft tissue infections, and locoregional nodular panniculitis [2,5,6]. The latter entity has been described in elderly patients with diabetes who have lower extremity anatomic changes and local skin injury [5].

Chronic wounds, particularly venous leg ulcers, are frequently colonized by *P. aeruginosa*. In a study of 754 patients with chronic venous leg ulcers, 84.3% had culture-positive ulcers, with *P. aeruginosa *among the most commonly identified alert pathogens [7]. In patients with recurrent venous leg ulcers, *P. aeruginosa *prevalence has been reported to be as high as 72.7% [8]. Persistence of *P. aeruginosa *in venous ulcers has been documented over years in individual patients and is associated with prolonged wound duration [9]. Distinguishing between colonization and clinically significant infection remains a persistent challenge [10,11].

We present the case of a 92-year-old nursing facility resident with multiple comorbidities who presented with acute green discoloration of his left foot, illustrating the clinical challenge of distinguishing *Pseudomonas* colonization from invasive infection and the importance of multidisciplinary management.

## Case presentation

A 92-year-old male presented to the ED because of concern that his left foot appeared noticeably green. The discoloration was first noticed by the nursing staff during routine care. The patient reported that he had last examined his left foot two days earlier during a dressing change, at which time no discoloration had been noted. Associated symptoms included left foot pain and swelling. He denied any injury or stepping on anything. He also denied difficulty with ambulation, fever, chills, or any new complaints aside from his chronic osteoarthritis. He ambulated with the assistance of a walker.

His past medical history was significant for chronic obstructive pulmonary disease, hypertension, type 2 diabetes mellitus (most recent hemoglobin A1c, 8.5%), stage 3a chronic kidney disease (CKD), chronic venous insufficiency, a history of deep venous thromboses treated with rivaroxaban, and chronic osteoarthritis.

**Figure 1:**
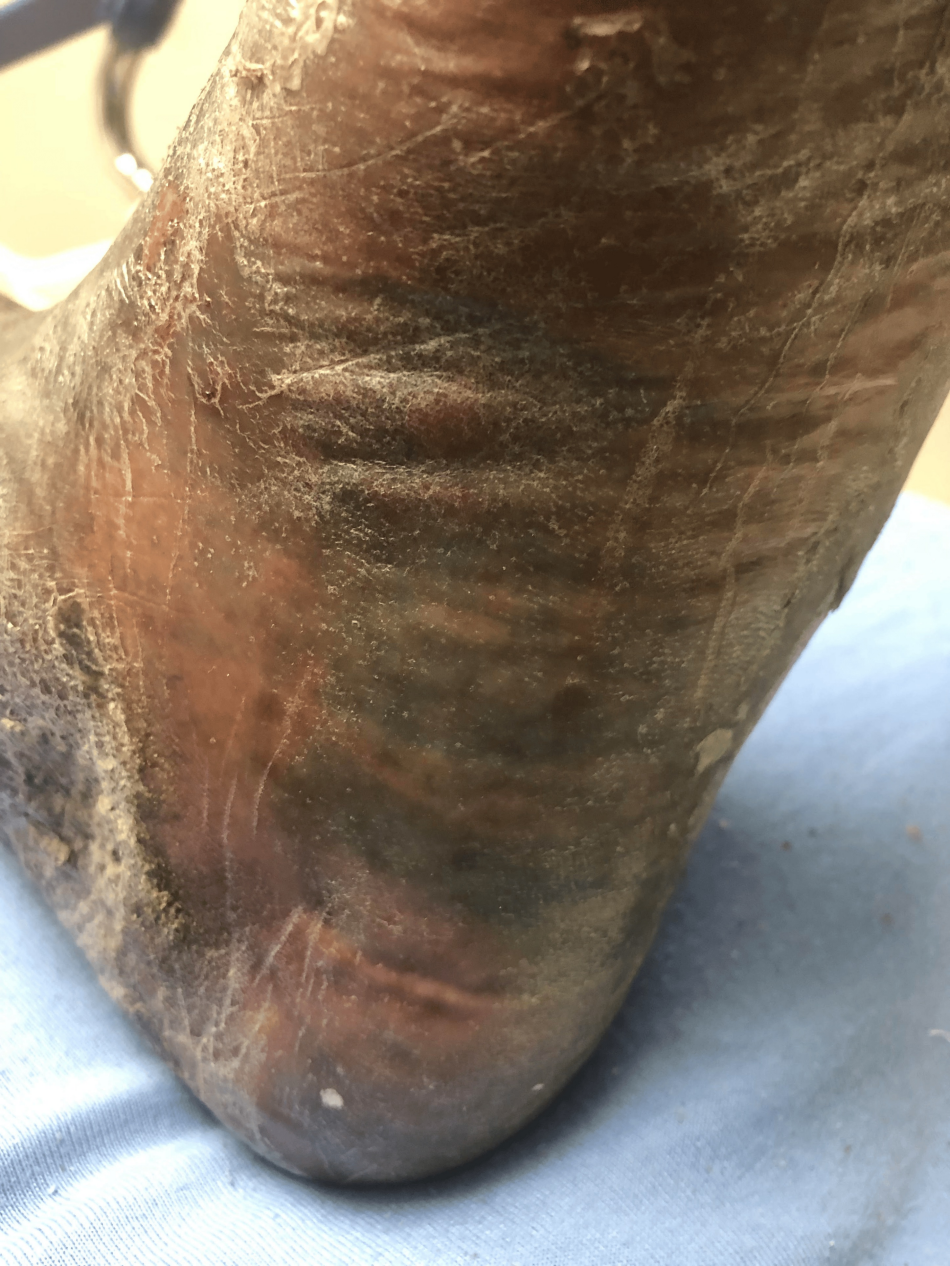
Close-up view of the plantar surface of the left foot highlighting the green-pigmented lesions in greater detail.

On examination, the initial vital signs were as follows: blood pressure, 168/86 mmHg; heart rate, 88 beats/min; respiratory rate, 18 breaths/min; temperature, 98.7°F (37.1°C); and oxygen saturation, 98% on room air. The patient was afebrile and hemodynamically stable. Examination of the left foot revealed striking green discoloration of the plantar surface (Figure 1).

**Figure 2:**
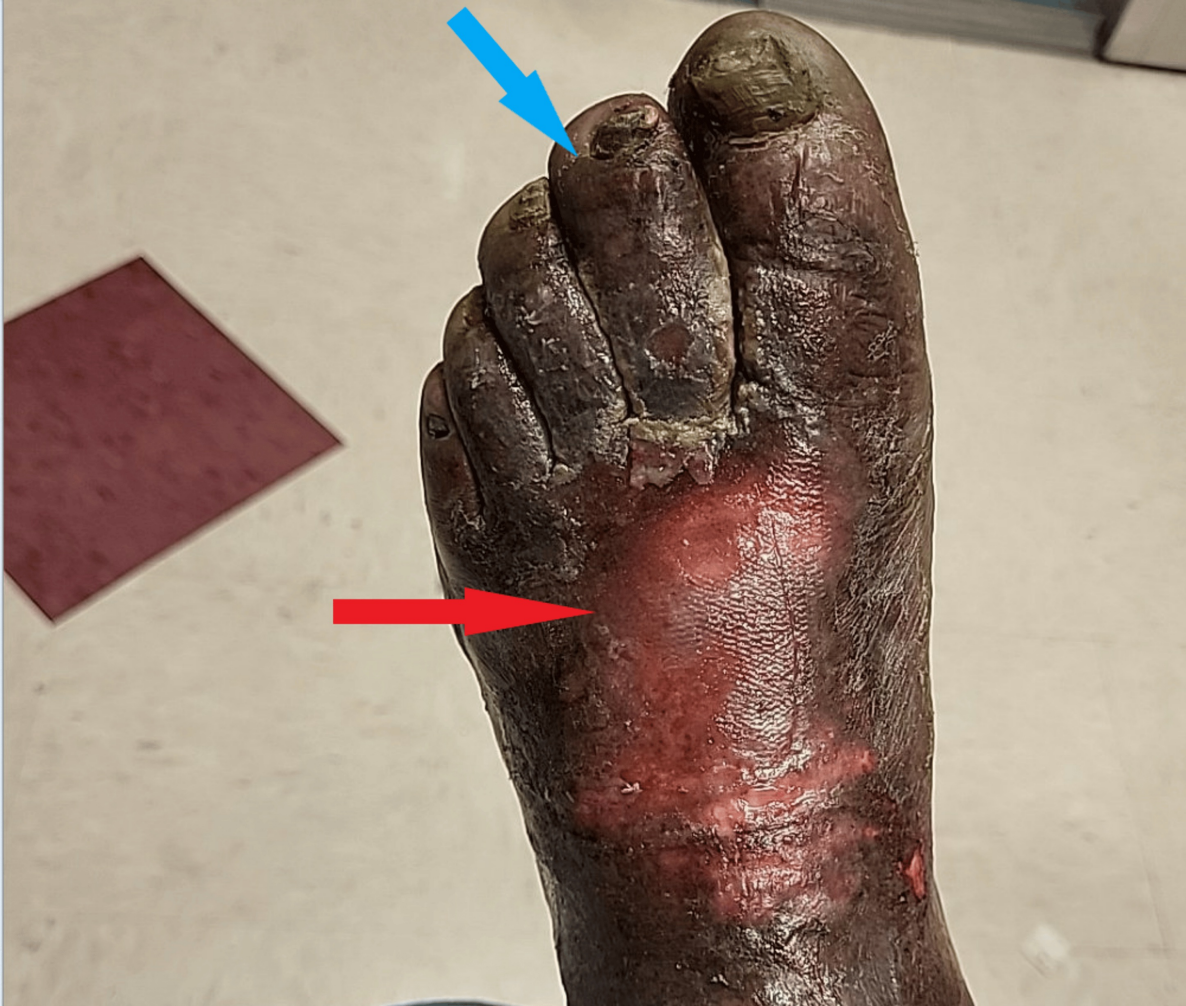
Clinical photograph of the superficial skin lesion on the dorsum of the left foot with inflammatory erythema (red arrow). The left foot, including the toes (blue arrow), is swollen.

Bilateral lower extremity edema and stasis dermatitis were present. Chronic skin hyperkeratosis was noted on both lower extremities, and onychomycosis of the toenails was observed. A superficial skin laceration was identified on the dorsal surface of the left foot, which had been managed with dressing changes at the nursing facility (Figure 2).

Capillary refill was less than two seconds. No other ulcers, purulence, crepitus, or areas of necrosis were identified.

Laboratory evaluation revealed no leukocytosis but a mildly elevated absolute neutrophil count. It also demonstrated mild normocytic anemia. A comprehensive metabolic panel was notable for an elevated blood urea nitrogen level and a creatinine level of 1.61 mg/dL, with an estimated glomerular filtration rate of 40 mL/min/1.73 m², consistent with acute kidney injury. His baseline creatinine level was 1.3 mg/dL. Serum lactate was normal. His most recent hemoglobin A1c was 8.5% (Table 1).

**Table 1:** Laboratory results summary.

Laboratory test	Result	Reference range
WBC count	9.8 × 10⁹/L	4.8-10.8 × 10⁹/L
Hemoglobin	10.7 g/dL	14.0-18.0 g/dL
Absolute neutrophil count	8.0 × 10⁹/L	1.4-6.5 × 10⁹/L
Creatinine	1.61 mg/dL (baseline, 1.30 mg/dL)	0.50-1.40 mg/dL
Blood urea nitrogen	69 mg/dL	8-21 mg/dL
Estimated glomerular filtration rate	40 mL/min/1.73 m²	≥60 mL/min/1.73 m²
Glucose	170 mg/dL	75-110 mg/dL
Lactate	1.2 mmol/L	0.5-2.0 mmol/L
Hemoglobin A1c	8.50%	4.7-5.6%

**Figure 3:**
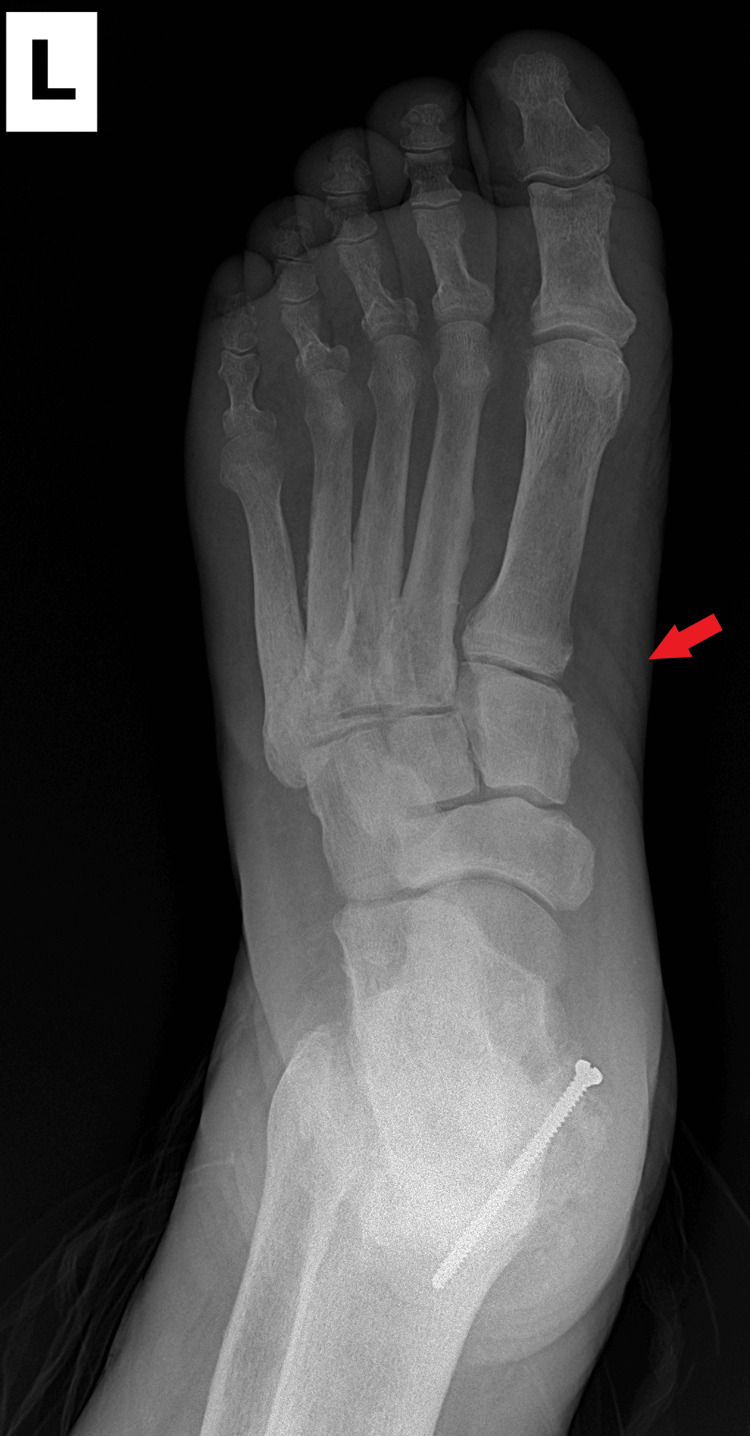
Anteroposterior radiograph of the left foot demonstrating soft tissue edema in the region corresponding to the area of green discoloration noted on physical examination (red arrow). An orthopedic screw is visualized in the medial malleolus.

**Figure 4:**
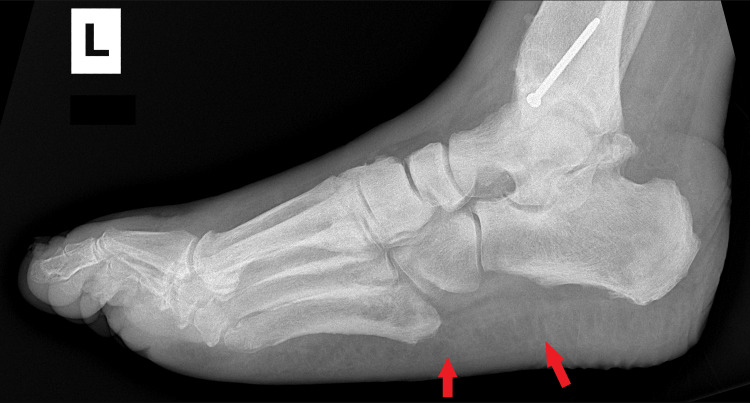
Lateral radiograph of the left foot demonstrating soft tissue edema in the region corresponding to the area of green discoloration noted on physical examination (red arrows). An orthopedic screw is visualized in the medial malleolus.

Radiography of the left foot demonstrated diffuse soft tissue edema and a surgical screw in the medial malleolus, without acute fracture or malalignment (Figures 3, 4).

In the ED, the patient received normal saline and empiric IV antibiotics with vancomycin and cefepime because of concern for possible soft tissue infection. He was admitted for observation and further workup, and an Infectious Diseases consultation was requested. The Infectious Diseases evaluation determined that the patient had developed excoriations on his left foot secondary to foot swelling caused by ill-fitting shoes, with superimposed cellulitis. The green discoloration was attributed to presumed pyocyanin production by colonizing *P. aeruginosa *on the skin surface, whereas the surrounding erythema and swelling were consistent with secondary bacterial cellulitis. Notably, microbiological confirmation of *P. aeruginosa *was not obtained in this case. This uncertainty limited the diagnostic interpretation of the findings. The diagnosis of presumed *P. aeruginosa *colonization was based on the clinical findings, specifically the acute green discoloration, lesion morphology, and clinical context. The antibiotics were transitioned to oral cephalexin 1,000 mg every 12 hours, with the addition of oral doxycycline 100 mg every 12 hours.

Wound care was consulted and recommended petrolatum-bismuth tribromophenate gauze dressings for the left foot, a urea-based moisturizing cream for the surrounding xerotic skin, and elevation of the left foot. Acetic acid soaks were also initiated as an adjunctive measure for the presumed *Pseudomonas* colonization.

**Figure 5:**
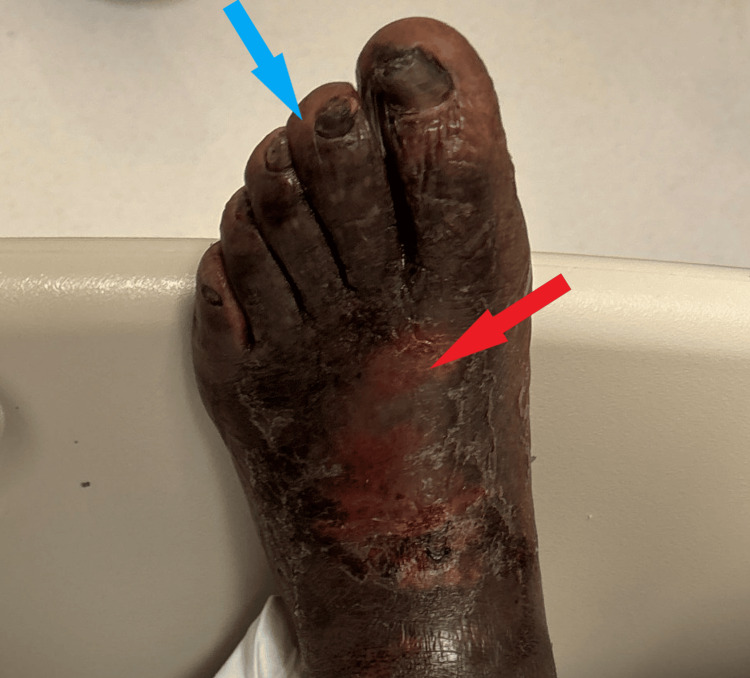
Clinical photograph of the dorsum of the left foot two days after initiation of treatment. The swelling of the left foot, including the toes (blue arrow), and the area of inflammatory erythema at the skin lesion on the dorsum of the foot (red arrow) were reduced.

Within two days, the patient demonstrated clinical improvement with resolution of left foot pain and improvement in the cellulitis. The swelling of the left foot and the area of inflammatory erythema at the skin lesion on the dorsum of the foot were reduced (Figure 5).

He remained afebrile with stable vital signs throughout the admission. Blood cultures obtained on admission remained sterile. Given the favorable clinical trajectory, IV cefepime was discontinued, and the patient was transitioned to oral cephalexin 1,000 mg every 12 hours, consistent with the Infectious Diseases Society of America (IDSA) guidelines recommending oral beta-lactam agents active against streptococci for typical cellulitis [12]. The WBC count remained within the normal range throughout the hospitalization and was 9.8 × 10⁹/L on the day of discharge.

Secondary issues addressed during the admission included acute kidney injury, with improvement in creatinine from 1.61 to 1.19 mg/dL, likely reflecting improved hydration status. Diabetes was managed with a consistent-carbohydrate diet and correctional insulin. The patient remained afebrile, hemodynamically stable, and without new hospital-acquired complications or falls throughout the admission. He was discharged to his nursing facility with a wound care referral and instructions to follow up with his primary care physician in one to two weeks.

A timeline of the clinical course is provided in Table 2.

**Table 2:** Timeline for the clinical course.

Timeline	Clinical events
Two days before presentation	No discoloration of the left foot was noted during routine dressing changes.
Day 0 - presentation to the ED	Acute green discoloration of the plantar surface of the left foot was noted. Laboratory studies showed a creatinine level of 1.61 mg/dL. The WBC count was within the normal range at 9.8 × 10⁹/L, with an absolute neutrophil count of 8.0 × 10⁹/L. Antibiotic therapy with cefepime and vancomycin was initiated.
Day 1	Treatment with acetic acid soaks was initiated.
Day 2 - day of discharge	Antibiotic therapy was transitioned to cephalexin and doxycycline. Laboratory studies showed a creatinine level of 1.19 mg/dL. The WBC count remained within the normal range at 9.8 × 10⁹/L. Reduction in pain, swelling, and erythema at the dorsal skin lesion of the left foot was noted.

## Discussion

This case illustrates the clinical dilemma of green skin discoloration in an elderly patient with multiple comorbidities predisposing to both *Pseudomonas* colonization and secondary bacterial infection. The striking green discoloration of the plantar surface of the foot was consistent with pyocyanin, the hallmark blue-green phenazine pigment produced by *P. aeruginosa* [1]. Pyocyanin production is an intricate quorum-sensing-driven process, and its visible accumulation on skin surfaces may reflect a significant colonizing bacterial burden [3]. However, the presence of green discoloration alone does not establish invasive infection. According to the International Working Group on the Diabetic Foot/IDSA 2023 guideline and its accompanying systematic review, blue-green discoloration, clinical assessment, and a characteristic grapefruit-like odor demonstrated low sensitivity (32%) and positive predictive value (18%) but high negative predictive value (92%) and reasonable specificity (84%), using culture as the reference standard. These findings suggest that such clinical signs are more useful for ruling out than ruling in *Pseudomonas* involvement in soft tissue diabetic foot infections [13,14].

The central clinical challenge in this case was distinguishing between *Pseudomonas* colonization of the skin, a common finding in patients with chronic venous insufficiency and stasis dermatitis, and true invasive soft tissue infection. Stasis dermatitis is a chronic inflammatory skin disease of the lower extremities that occurs as the cutaneous manifestation of venous hypertension, typically in older individuals [15]. The associated edema, skin barrier disruption, and chronic inflammatory changes create an environment highly conducive to bacterial colonization [7,10]. In a systematic review of chronic wound infection diagnosis, the reference standard was deep tissue biopsy culture, whereas surface swab cultures had limited value in distinguishing colonization from infection [11,16]. The IDSA and American Society for Microbiology guidelines similarly emphasize that surface cultures of chronic wounds represent colonizing microbes that cannot be readily differentiated from true pathogens [11]. Elderly patients residing in nursing facilities with comorbidities such as diabetes mellitus, chronic venous insufficiency, and CKD represent a vulnerable population for *Pseudomonas *skin colonization and secondary infection. Classic signs of infection, such as pain, heat, redness, swelling, and purulence, may be absent or attenuated in chronic wounds [10]. Signs of critical colonization, including delayed healing, unexpected pain, abnormal odor, and discolored granulation tissue, have been proposed as alternative indicators [10]. The reference standard for diagnosing infection in chronic wounds remains deep tissue biopsy culture, as surface swab cultures primarily reflect colonizing flora [11,16]. In this patient, the absence of systemic inflammatory signs (afebrile, normal lactate, and hemodynamically stable), negative blood cultures, and the superficial nature of the green discoloration favored colonization over invasive *Pseudomonas* infection. However, the Infectious Diseases team identified superimposed cellulitis arising from excoriations caused by ill-fitting footwear, which served as a portal of entry for skin flora. This distinction was clinically important, as it guided the antibiotic regimen toward standard cellulitis therapy rather than prolonged antipseudomonal coverage.

Given that *Pseudomonas* is a waterborne pathogen, potential sources of exposure, including hydrotherapy or specific washing practices at the nursing facility, were considered; however, no such exposures were identified in this patient. Tight-fitting footwear likely contributed to *Pseudomonas* colonization by creating a warm, moist environment. The Infectious Diseases specialist considered that the shoes caused friction, leading to skin breakdown. The moisture-rich environment also resulted in skin maceration, reducing the protective function of the skin barrier. This provided a surface for bacterial adhesion and colonization.

Several predisposing factors in this patient merit discussion. Chronic venous insufficiency with stasis dermatitis is a well-recognized risk factor for cellulitis, with lower extremity edema, venous insufficiency, and toe web abnormalities all identified as predisposing conditions in the IDSA guidelines [12]. Poorly controlled diabetes mellitus (hemoglobin A1c, 8.5%) impairs immune function and wound healing through multiple mechanisms, including hyperglycemia-induced leukocyte dysfunction, increasing susceptibility to both colonization and infection [5,17]. The patient’s residence in a nursing facility further compounded his risk, as institutional settings are associated with increased exposure to health care-associated organisms, including *P. aeruginosa* [7]. The combination of chronic venous insufficiency, diabetes, CKD, advanced age, and institutional residence created a convergence of risk factors that predisposed this patient to both *Pseudomonas* colonization and secondary cellulitis.

The management approach in this case reflected a pragmatic balance between treating the confirmed cellulitis and addressing the *Pseudomonas *colonization. Empiric broad-spectrum coverage with vancomycin and cefepime was initiated in the ED because of diagnostic uncertainty. Following the Infectious Diseases evaluation, the antibiotic regimen was narrowed. The transition from IV cefepime to oral cephalexin was consistent with evidence supporting early oral step-down therapy for cellulitis. A systematic review and meta-analysis found no significant difference in treatment failure between short-course (five days or fewer) and longer-course antibiotic therapy for uncomplicated cellulitis [18]. The IDSA guidelines recommend a treatment duration of five days for uncomplicated cellulitis, with extension if the infection has not improved [17]. The total seven-day course in this patient was appropriate given his multiple comorbidities and the superimposed nature of the infection.

Cellulitis is predominantly caused by beta-hemolytic streptococci, with *Staphylococcus aureus* playing a secondary role in cases involving penetrating trauma, injection drug use, or purulent drainage [19]. In this patient, the absence of purulence and the mechanism of injury (excoriations from ill-fitting shoes) were consistent with a streptococcal etiology, supporting the choice of cephalexin as step-down therapy [12,19]. The addition of doxycycline provided additional coverage for potential methicillin-resistant* S. aureus* (MRSA), given the patient’s nursing facility residence and chronic wound, consistent with IDSA recommendations for purulent infections or MRSA risk factors [12].

Acetic acid soaks were employed as an adjunctive topical measure targeting *Pseudomonas *colonization. In a prospective randomized controlled trial, 1% acetic acid dressings applied to chronic wounds infected with *P. aeruginosa* achieved elimination of *Pseudomonas* in a mean of 4.5 days in the acetic acid group, compared with 11.5 days in the control group [20].

This case underscores the importance of a multidisciplinary approach involving Infectious Diseases and wound care in managing complex lower extremity infections in elderly patients. The Infectious Diseases consultation was instrumental in distinguishing colonization from infection and guiding appropriate antibiotic therapy, while the wound care consultation addressed the underlying skin barrier dysfunction and provided topical management strategies.

## Conclusions

This case describes a 92-year-old nursing facility resident with multiple comorbidities who presented with acute green discoloration of the left foot, consistent with a presumptive clinical diagnosis of *P. aeruginosa *colonization with pyocyanin production and superimposed cellulitis arising from excoriations caused by ill-fitting footwear. The presence of chronic venous insufficiency, diabetes, and impaired skin integrity increased the complexity of the case. The clinical course highlights the importance of distinguishing *Pseudomonas *colonization from invasive infection when microbiological confirmation is unavailable, allowing appropriate antimicrobial de-escalation from empiric broad-spectrum therapy to targeted treatment for cellulitis. The patient responded well to systemic antibiotics, acetic acid soaks, and wound care and was discharged to his nursing facility.

Green skin discoloration, while alarming, should prompt careful clinical assessment rather than reflexive broad-spectrum antibiotic escalation. A multidisciplinary approach incorporating Infectious Diseases and wound care, together with optimization of footwear, edema management, glycemic control, and skin barrier integrity, may help improve outcomes and reduce recurrence in elderly patients with multiple comorbidities.
